# Application of Advanced Platelet-Rich Fibrin Plus in Oral Wound Healing and Pain Management: A Systematic Literature Review

**DOI:** 10.3390/jfb16100360

**Published:** 2025-09-26

**Authors:** Marek Chmielewski, Andrea Pilloni, Paulina Adamska

**Affiliations:** 1 Private Dental Practice, 81-881 Sopot, Poland; 2 Section of Periodontics, Department of Oral and Maxillo-Facial Sciences, Sapienza Unviersity of Rome, 00-185 Rome, Italy; andrea.pilloni@uniroma1.it; 3 Department of Oral Surgery, Medical University of Gdansk, 80-211 Gdansk, Poland

**Keywords:** advanced platelet-rich fibrin, A-PRF+, autografts, dentistry, growth factors, wound healing

## Abstract

**Background:** The growing interest in the field of platelet-rich fibrins has led to the development of novel generations of these concentrates, with one of the newest additions being advanced platelet-rich fibrin plus (A-PRF+). The updated centrifuge protocol utilized for the preparation of A-PRF+ has been shown to provide blood clots with more white blood cells and growth factors trapped in the fibrin matrix, presenting a more homogenous distribution. The objective of this study was to assess the available randomized clinical trials (RCTs), in order to evaluate the effects that the addition of A-PRF+ can have on postoperative quality of life and soft tissue healing after dental surgery. **Materials and Methods:** To perform a systematic review based on high-quality results, only RCTs were taken into consideration. The search included articles published between 1 January 2014 and 31 December 2024, indexed in the PubMed, Cochrane, Library, Embase, Scopus, and Google Scholar databases. Nine full texts were finally acquired after the screening of articles, from which relevant data were extracted. **Results:** A-PRF+ positively influenced the postoperative quality of life in patients. The subjective analysis of pain and its management via painkiller intake indicated that, in general, the addition of A-PRF+ into protocols results in less pain, pain that lasts for a shorter time, and pain that is more easily managed through medication, as a lower number of pills was needed to restore comfort. Furthermore, the occurrence of facial swelling and trismus was also reported to be lower in the A-PRF+ groups. As for soft tissue healing, A-PRF+ significantly enhanced the epithelialization process, total wound area reduction, and inflammation in the surrounding tissues. This positive effect was most noticeable at 7- and 14-day follow-ups. The addition of A-PRF+ also had a positive effect on postoperative bleeding by significantly reducing the bleeding time, providing benefits for patients undergoing antiplatelet drug therapy in particular. **Conclusions:** The addition of A-PRF+ into the surgical protocol can positively enhance the patient’s quality of life, reduce the need for postoperative medication, and improve the patient’s confidence by reducing potential swelling and trismus. A-PRF+ also positively influences soft tissue wound healing, further enhancing the postoperative well-being of patients, and provides an excellent hemostatic effect even in patients that are on antiplatelet drug therapy.

## 1. Introduction

The quest to identify a blood-derived additive which is capable of enhancing post-surgical healing by leveraging the biological potential of the human body has been a long-standing objective for numerous clinicians. The initial breakthrough in this endeavor was the discovery of platelet-rich plasma (PRP) [1,2]. The subsequent surge in interest, accompanied by the observation of favorable clinical outcomes and the broadening scope of adoption, led to the development of a second-generation platelet derivative: platelet-rich fibrin (PRF) [3,4,5,6]. Subsequent to this, a third generation of second-generation platelet concentrates was introduced, including advanced platelet-rich fibrin (A-PRF), advanced platelet-rich fibrin plus (A-PRF+), leukocyte- and platelet-rich fibrin (L-PRF), injectable platelet-rich fibrin (I-PRF), titanium-prepared platelet-rich fibrin (T-PRF), and autologous fibrin glue (AFG). Factors distinguishing these concentrates include parameters such as the time, centrifugal force, and tube material (glass or plastic tubes) utilized during the centrifuging process [7,8,9,10]. One of the newest of the aforementioned concentrates is A-PRF+, which was introduced in 2017.

To obtain A-PRF+, fresh venous blood must be drawn from the patient prior to centrifugation. The most common and convenient sites for venipuncture are the median cubital vein or veins in the antecubital fossa, due to their accessibility and relatively large diameter. If a suitable vein cannot be located, non-invasive near-infrared vein finders can be used to identify a viable location for venipuncture [11]. The requirement for A-PRF+ preparation is 10 mL of blood per portion. It is imperative to emphasize that the blood must be drawn into a blood collection tube without any anti-coagulant agents (i.e., sodium citrate) which, when present in blood collection tubes, prevent clotting and therefore make it impossible to obtain a platelet-rich fibrin preparation. Subsequent to collection, the tubes are placed within the centrifuge, with the program set at 1300 rpm for a duration of 8 min [12]. A reduction in time is observed when compared with the standard A-PRF protocol (i.e., 14 min for A-PRF vs. 8 min for A-PRF+), yet the speed of the centrifuge remains unaltered (both stay at 1300 rpm) [9,10,13]. Subsequent to the completion of centrifugation, the red-blood-cell-rich fraction is physically separated from the fibrin clot, typically by cutting with sterile scissors. The prepared A-PRF+ can then be applied directly to the wound, placed onto a metal strainer and pressed with the lid to form membranes, or placed into molds to obtain A-PRF+ corks.

Platelet-rich fibrin has been shown to contain a variety of cells, including platelets, monocytes, lymphocytes, and stem cells. In addition, it has been demonstrated to contain growth factors such as platelet-derived growth factor AB, transforming growth factor-β, fibroblast growth factor, epidermal growth factor, growth factor, hepatocyte growth factor, insulin-like growth factors 1 and 2, matrix metalloproteinases, and interleukins. These are all linked with the activation and/or management of the destroyed tissue and inducing tissue healing, tissue proliferation, and angiogenesis [13,14,15,16,17,18]. Compared with standard PRF, advanced PRF—introduced by Ghanaati et al. [13] through adjustment to the centrifuge protocol—improves the distribution of T-lymphocytes, B-lymphocytes, stem cells, and monocytes in the proximal 25–30% of the obtained clot while, at the same time, providing a more homogenous platelet distribution in the matrix scaffolding. The even distribution of platelets leads to even extraction of growth factors, cytokines, chemokines, and primary healing molecules, thus enabling homogenous activation of other cells trapped in the fibrin matrix. The subsequent development of A-PRF+ by Fujioka-Kobayashi et al. [12] demonstrated that a reduction in the centrifuge time can positively influence the obtained PRF by increasing the release of platelet-derived growth factor, transforming growth factor-β, epidermal growth factor, and insulin-like growth factor, affecting the cellular response at the surgical site.

In our study, we aim to address the following research question: What are the impacts of A-PRF+ on postoperative pain levels and soft tissue healing, compared to standard protocols, in oral surgery?

## 2. Materials and Methods

This project was registered in the International Prospective Register of Systematic Reviews (PROSPERO) and granted the number CRD42023449648. The PRISMA (Preferred Reporting Items for Systematic Reviews and Meta-Analyses) guidelines were followed. The research question for this systematic review was: Does A-PRF+ enhance the quality of life and soft tissue healing in patients after surgical procedures? The PICO framework (Population, Intervention, Comparison, Outcome) was applied as follows:

- Population (P): patients undergoing dental treatment utilizing advanced platelet-rich fibrin plus (A-PRF+).
- Intervention (I): oral surgical procedures involving soft tissue manipulation, in which A-PRF+ was applied.
- Comparison (C): standard treatment approaches using A-PRF+, alone or in combination with other forms of PRF, compared with conventional regenerative techniques.
- Outcome (O): evaluation of soft tissue regeneration and patient quality of life following surgery, including the assessment of pain, swelling, and other postoperative symptoms over time.

### 2.1. Search Strategy

To comprehensively evaluate the available literature on the addition of A-PRF+ into dental surgical procedure protocols, an extensive database search including PubMed, Cochrane Library, Embase, Scopus, and Google Scholar was conducted. To ensure replicability of the conducted database search, the following MeSH terms were used: ‘platelet-rich fibrin’, ‘PRF’, ‘autografts’, ‘dentistry’, ‘growth factors’, and ‘wound healing’. To increase the search sensitivity and target articles focused on A-PRF+, relevant keywords in titles and abstracts were included: ‘advanced platelet-rich fibrin+’, ‘A-PRF+’, ‘dental surgery with A-PRF+’, and ‘A-PRF+ healing’. If possible, filtering for randomized clinical trials was performed.

### 2.2. Inclusion and Exclusion Criteria

To screen the retrieved literature, the following inclusion criteria were used: (1) studies using advanced platelet-rich fibrin plus (A-PRF+) in dental surgical procedures focused on soft tissues and/or postoperative patient quality of life; (2) studies conducted in humans; (3) randomized clinical studies (RCTs) with a minimum of 10 patients enrolled; (4) studies written in English; and (5) studies with full-text available at the time of conducting the systematic review.

The exclusion criteria were: (1) studies that did not utilize A-PRF+; (2) studies that focused only on hard tissue management without consideration of postoperative soft tissue behaviors; (3) studies that did not have a sufficient number of participants (less than 10); (4) studies written in a language other than English; (5) pre-prints and commentaries; or (6) manuscripts in press that were not available in full at the time the search was performed.

### 2.3. Data Screening

The search included manuscripts published up until 31 December 2024. The initial screening process involved reading the titles of relevant articles to identify those that met the inclusion criteria, and was conducted by the first author. Further critical assessment of identified publications was carried out by the first author under the supervision of the third author. The search, screening, and data extraction process was monitored by the second investigator, whose opinion prevailed in the event of any disagreement. All duplicates were excluded. The articles were checked a second time for compliance with the inclusion criteria by reading their abstracts. Finally, the full-text articles were obtained and subjected to analysis in accordance with the established selection criteria. Finally, a total of nine publications were deemed eligible for inclusion in this review.

### 2.4. Data Extraction

After the search and screening, data from each of the articles that were selected for inclusion in the study were divided according to general topic categories (postoperative pain, swelling and trismus occurrence, wound healing, and postoperative control of bleeding). The results from each study were extracted and presented in the table corresponding with one of the aforementioned topics. Moreover, basic information (first author, date of publication, research country, procedure, number of patients, follow-up period, complications) from each of the studies were extracted and included in the general table to provide an introductory overview of all of the included studies. All tables were created using the Microsoft Excel spreadsheet program by the first author, then verified by the second and third authors separately to ensure that the data were extracted correctly.

### 2.5. Quality Assessment

The risk of bias was assessed using the Revised Cochrane risk of bias tool for randomized trials (RoB 2) [19,20]. These procedures were performed by the third and first authors and, in the case of a disagreement, a consensus reading was carried out.

## 3. Results

**Figure 1:**
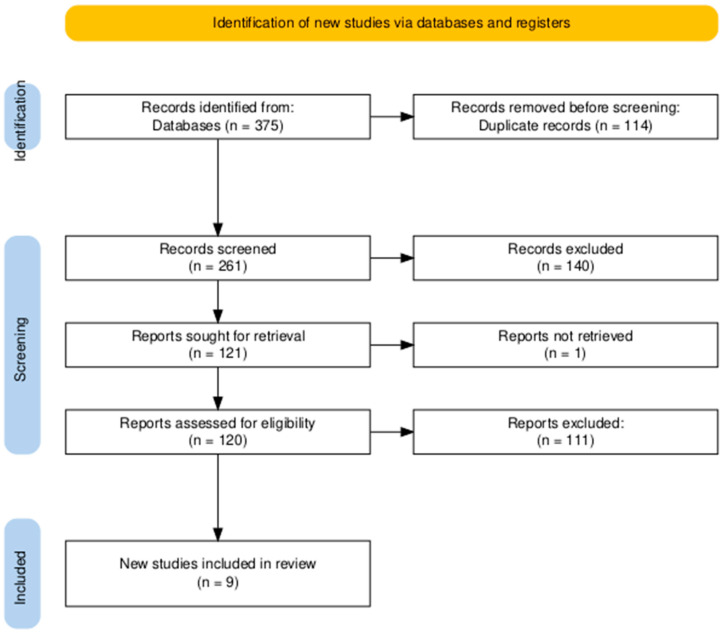
PRISMA flowchart.

The initial screening process was conducted by reading the titles of relevant articles in order to identify those that satisfied the inclusion criteria. This initial search of two databases yielded a total of 375 articles, of which 114 articles were duplicates. After reading abstracts, a total of nine [21,22,23,24,25,26,27,28,29] articles were found to be suitable for inclusion in the systematic review. The PRISMA flowchart is presented in Figure 1. The general extracted data, including authors, date, place of research, procedure, patient count, A-PRF+ preparation protocol, follow-up times, and complications are presented in Table 1.

### 3.1. Effects of A-PRF+ on Postoperative Pain and Analgesic Use

A total of five studies that evaluated the VAS pain score were identified [25,26,27,28,29], two of which additionally measured subsequent analgesic usage [27,29]. In general, patients who received A-PRF+ exhibited reduced postoperative pain and decreased postoperative analgesic usage. A-PRF+ was predominantly compared with sterile saline irrigation and blood clot formation.

Pereira et al. [25] and Sousa et al. [27] both measured the VAS pain score as a median (with interquartile range—IQR) at each follow-up visit. Sousa et al. compared an A-PRF+ group against a saline control. In both groups, the median VAS pain score reached 0 at day 14. The difference in pain perception was found to be statistically significant on day 2, in favor of the A-PRF+ group. On day 7, the test group reported no pain while the control group reported some discomfort, although the difference was not statistically significant. Pereira et al. compared a test group with a saline control group. In contrast to Sousa et al., Pereira et al. did not observe a favorable outcome with A-PRF+ in terms of a decrease in the VAS pain score. On day 2, the control group exhibited a lower median and IQR than the A-PRF+ group; however, these differences were not statistically significant. On day 7, the IQR of the control group was lower than that of the A-PRF+ group, although this difference was not statistically significant either. At day 14, both the test and control groups had equal median and IQR values [25,27].

Other interpretations of the VAS pain score have been presented by Soto-Peñaloza et al. [26] and Yüce et al. [29]. Both papers used saline as a control and presented results on the VAS scale (0–10). Yüce et al. reported the average VAS pain scores of patients at each follow-up visit with respect to the baseline pain score on the day of surgery; namely, for day 1 (control 7.25 ± 1.02, test 5.2 ± 1.06), day 3 (control 7.05 ± 1.23, test 2.25 ± 0.64), and day 5 (control 5.9 ± 0.91, test 0.8 ± 0.62). The mean values obtained for the test and control groups of 9 ± 0.91 (on the first postoperative day) and 0.8 ± 0.62 (on the seventh postoperative day) were compared. The A-PRF+ group demonstrated a statistically significant reduction in postoperative pain when compared with the control group (*p* < 0.05); furthermore, this finding was consistent across all postoperative visits. In contrast, Soto-Peñaloza et al. reported the average VAS pain score at the 7-day follow-up. The control group exhibited a higher postoperative pain score (2.07 ± 1.63), when compared to the test group (1.27 ± 0.85); however, this difference was not statistically significant. Furthermore, both Yüce et al. and Soto-Peñaloza et al. reported on analgesic usage after 7 days. Yüce et al. noted a statistically significant decrease in analgesic usage for the test group (3.6 ± 1.19) compared with the control group (13.05 ± 1.32). Even though Soto-Peñaloza et al. did not provide exact numbers for total analgesic consumption, the reported trend showed a similar decrease in analgesic usage between the groups [26,29].

Yewale et al. [28] utilized a different approach in their VAS pain score comparison, focusing on the percentage of patients experiencing different VAS score ranges. Their findings revealed that the test and control groups exhibited similar outcomes, with the control group demonstrating slightly higher percentages of mild (30%) and lower percentages of moderate (70%) pain. Conversely, the test group exhibited slightly lower percentages of mild (20%) and higher percentages of moderate (80%) pain. All of these findings are comprehensively summarized in Table 2.

### 3.2. Effect of A-PRF+ on Postoperative Swelling and Trismus

A total of four studies evaluated either swelling, edema, or trismus in their clinical outcomes. Of these studies, one reported on trismus and three on swelling and edema. The results presented for swelling differed in each of the three manuscripts, due to the varying manner in which the results were presented [21,25,26,28].

Alhaj et al. [21] measured the swelling in centimeters and calculated it as the mean of the distance between the lateral corner of the eye and the angle of the mandible, tragus, and the outer corner of the mouth, as well as between the tragus and soft tissue pogonion. Compared to the baseline, the median facial swelling increased in both groups at day 2. In the A-PRF+ group, this increase was smaller (0.26 cm) than in the control group (0.36 cm). By day 7, the swelling in the A-PRF+ group had completely subsided, while it remained in the control group (0.11 cm); however, these differences were not statistically significant. Although Pereira et al. [25] also measured the median facial swelling, it was based on reports from the VAS scale, not clinical measurements. The median edema score was comparable between the control and test groups on both the third and seventh days; however, a distinction between the test and control groups emerged in the interquartile range, with control and test group IQRs at the day 3 follow-up of 0.25–3.75 and 0.25–4.5. The difference between the groups remained at day 7, with control and test group IQRs of 0.0–1.75 and 0.0–2.75, respectively.

In contrast to the focus on the severity of swelling in previous studies, Yewale et al. [28] examined the occurrence of swelling in their study population by means of the visual appearance of swelling and patient’s feelings. The test group, which utilized A-PRF+, exhibited a significantly lower incidence of facial swelling (30% for the test group and 80% for the control group). Moreover, in cases where swelling did occur, patients in the test group reported that the edema had subsided by three days post-surgery. In contrast, the control group exhibited a subsidence of swelling after four days for 37.5% of patients and after five days for 62.5%.

The study by Soto-Peñaloza et al. [26] focused on the occurrence of trismus in the study population and measured the average occurrence (as a percentage of the study population) of subjective reduction in maximal mouth opening (MMO). For the patients who were administered A-PRF+, the occurrence of reduction in MMO was lower (68%) than in the control group (96%). Unfortunately, they did not evaluate the clinical significance of these results. All of these findings are comprehensively summarized in Table 3.

### 3.3. Effects of A-PRF+ Addition on Postoperative Wound Healing

A total of six studies that reported on postoperative wound healing after surgical procedures were identified. The main categories that were identified included changes in the total wound area (in mm^2^) [27], wound area reduction (in percentage) [25], visible epithelialization (in percentage) [27], and wound healing [22,23,25,26,29].

In the study conducted by Sousa et al. [27], postoperative wound healing was measured in terms of changes in the total wound area, wound area reduction, and visible epithelialization. The A-PRF+ test group was compared with a gelatin sponge control group. With regard to the total wound area, the initial average measurements were comparable between the control (122 ± 43.1 mm^2^) and test (121.4 ± 27.8 mm^2^) groups. At the day 2 follow-up, both the control (119 ± 41.6 mm^2^) and test (118 ± 30.8 mm^2^) groups exhibited comparable total wound areas. However, at the day 7 follow-up visit, the test group demonstrated a significant decrease in total wound area (77.3 ± 23.3 mm^2^) when compared with the control group (105.1 ± 33.4 mm^2^). This trend persisted at day 14 (74.5 ± 31.9 mm^2^ for the control group, 50.3 ± 16.6 mm^2^ for the test group) and day 30 (40 ± 17.2 mm^2^ for the control group, 11 ± 18.8 mm^2^ for the test group). At the final follow-up at day 90, complete healing of the palatal wounds was observed in both the control and test groups. Unfortunately, the clinical significance of the observed variations in total wound area was not assessed. With respect to the reduction in wound area, the initial follow-up at day 2 revealed comparable percentages between the control (2 ± 5.1%) and test (2.9 ± 10.7%) groups, though the test group exhibited slightly higher percentages. The difference between the groups widened at day 7 (12.9 ± 12.2% control group, 36.4 ± 12.2% test), day 14 (36.6 ± 20.4% control, 58.0 ± 14.2%), and day 30 (50.9 ± 14.3% control, 90.5 ± 14.6% test group). The results indicated statistical significance between the two groups at the aforementioned time points. At the final follow-up, complete healing was observed in both the control and test groups. The assessment of epithelialization in both groups at 2- and 7-day follow-ups indicated no difference between the groups, and no visible epithelialization was present. At day 14 (9.1% control group, 64.3% test group), the difference in the percentage of visible epithelialization was statistically significant between the groups, favoring A-PRF+. At day 30, this difference lost its statistical significance, and the two groups exhibited comparable outcomes (90.9% control group, 92.9% test group). At the final follow-up visit, the epithelialization of wounds was complete (100%) in both groups.

The remaining five studies employed a variety of methodologies for assessment of wound healing. Yüce et al. [29] measured wound healing using the Landry, Turnbull, and Howley (LTH) epithelial healing index. At days 7 (2.05 ± 0.69 for the control group, 3.65 ± 0.49 for the test group) and 14 (3.55 ± 0.6 for the control group, 4.8 ± 0.41 for the test group), the results obtained using the LTH index were superior for the A-PRF+ group. At both time points, the A-PRF+ group exhibited a statistically significant improvement. Giudice et al. [25] also presented their results in terms of a healing index; however, it differed from the one used by Yüce et al. [29]. At the one-week follow-up, the A-PRF+ group was the second best in their study with a mean index score of 1.0 ± 0.68, with L-PRF providing a slightly better result at 0.95 ± 0.5. The control group exhibited a mean score of 1.05 ± 0.6, while the hemostatic agent (HEM) group demonstrated a mean score of 1.18 ± 0.59. This trend persisted at the second follow-up visit in the second postoperative week, with mean scores of 0.15 ± 0.36 for L-PRF, 0.25 ± 0.49 for A-PRF+, 0.43 ± 0.5 for HEM, and 0.33 ± 0.53 for control. The differences between the groups were not statistically significant, although L-PRF and A-PRF+ generally performed better. Soto-Peñaloza et al. [24] measured the occurrence of inflammation at the post-surgical site at the 7-day follow-up. For the control group, the occurrence of inflammation around the postoperative area reached 100%, whereas it reached 80% for the A-PRF+ group; however, this difference was not statistically significant (*p* = 0.11). Brancaccio et al. [22] examined the percentage of incomplete healing at the follow-up visit (two weeks after surgery). Consistent with the study of Giudice et al. [19], the lowest percentage of incomplete healing was observed in the L-PRF group (15%), followed by the A-PRF+ group (22%). The hemostatic test group (38%) exhibited a higher percentage of incomplete healing, compared to the control group (30%). A statistically significant difference was observed between the L-PRF and control groups, as well as between the HEM group and the L-PRF and A-PRF+ groups. However, no statistically significant difference was observed between the L-PRF and A-PRF+ groups.

Pereira et al. [23] presented their results as medians with interquartile ranges. The outcomes of their study deviated from the previously cited ones, as A-PRF+ performed worse than the control. The most significant discrepancy was observed during the follow-up visits at day 3 (median 1.5 with IQR 0–5 for the control group, median 3 with IQR 0.25–5 for the test group) and day 7 (median 0 with IQR 0–2.75 for the control group and median 1 with IQR 0–2.75 for the test group). Subsequent visits revealed no significant difference in median score between the two groups; however, differences in the IQR were observed at days 30 and 90, favoring the control group. All of these findings are comprehensively summarized in Table 4.

### 3.4. The Effects of A-PRF+ on Postoperative Bleeding

A total of four articles measured the effects of A-PRF+ on postoperative bleeding. Two of the studies measured the hemostatic effect at 30 min after the surgery [22,23,25,26].

Brancaccio et al. [22] measured the percentage of patients with bleeding at 30 min post-tooth extraction surgery. The control group, which was treated with the standard procedure of wound cleansing with a saline solution and suturing, was contrasted with three test groups: Hemostatic plug, A-PRF+, and L-PRF. After a 30 min interval, the test group that utilized A-PRF+ exhibited the most favorable outcomes, with 2% of patients experiencing postoperative bleeding. This outcome was statistically significant when compared with the control group. The L-PRF test group exhibited slightly higher postoperative bleeding, with 5% of patients reporting this complication; this outcome was also statistically significant. The hemostatic plug test group demonstrated a lower rate of postoperative bleeding (12%) compared to the control group (20%). However, both the control and the hemostatic agent test groups exhibited significantly higher rates of postoperative bleeding compared to the other test groups. Similarly to Brancaccio et al., Giudice et al. [23] also examined the bleeding at 30 min post-surgery. Their presentation of results differed from that of Brancaccio et al., with Giudice et al. reporting results in odds ratio (OR) format. Similarly, the control group received wound rinsing with saline and sutures, and three test groups were examined: Hemostatic plug, A-PRF+, and L-PRF. The A-PRF+ group exhibited the most favorable outcomes, with an odds ratio of 0.1, followed by the L-PRF group with an odds ratio of 0.21. Notably, the A-PRF+ group demonstrated statistical significance. In contrast, the hemostatic agent test group exhibited suboptimal performance in comparison to the control group, with odds ratios of 0.57 vs. 0.25, respectively. A notable point is that both studies [22,23] were conducted in patients treated with antiplatelet drugs.

Pereira et al. [23] examined the persistence of postoperative bleeding during subsequent follow-up visits. At the initial visit (three days post-surgery), the median for the control group was 0.5 (with an IQR of 0–2) while, for the A-PRF+ group, the median was 1 (with an IQR of 0–2); however, this finding did not reach statistical significance. At subsequent follow-up visits, no bleeding was observed in either group. Finally, Soto-Peñaloza et al. [24] reported the percentage of bleeding, as reported by patients, up until the follow-up visit at 7 days. The test group using A-PRF+ reported less postoperative bleeding (28%) than the control group using saline rinsing and stitches (52%). This difference nearly reached statistical significance, with a *p*-value of *p* = 0.087. All of these findings are comprehensively summarized in Table 5.

### 3.5. Quality Assessment

**Figure 2:**
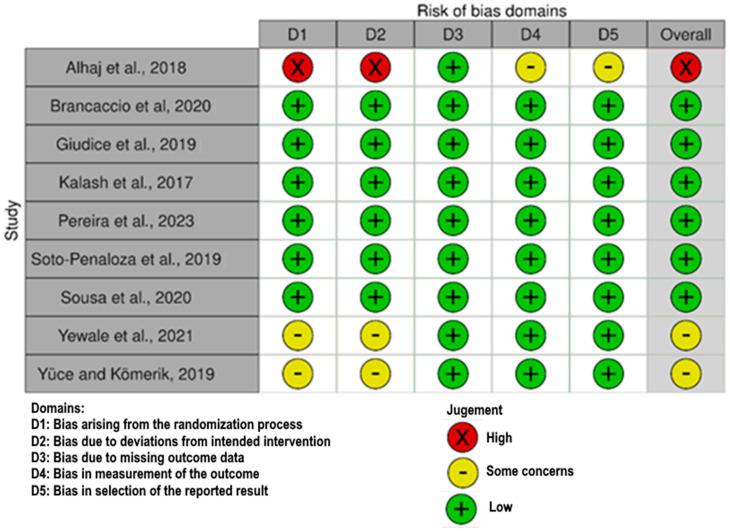
The risk of bias assessment using RoS 2 [21,22,23,24,25,26,27,28,29].

The risk of bias assessment results are presented in Figure 2.

## 4. Discussion

Since their inception, the utilization of platelet-rich blood derivatives has shown a consistent upward trend in the field of dentistry. As our understanding of the complexities of molecular interferences and their impacts on cellular pathways and the modulation of their responses deepens, the precise selection of appropriate centrifuge conditions to obtain optimal PRF variants has become crucial. Subsequent modifications have been developed with the aim of improving the quality of blood-derived products at both the macro- and micro-levels. These changes result in more cohesive substances which, in oral surgery, provide better retention effects in both hard and soft tissues within wounds and defects. In addition, they enable the more efficient extraction of growth factors and cells which, in turn, positively influence tissue regeneration and repair processes, enhance healing, shorten treatment times, and reduce the occurrence of adverse effects associated with surgery (e.g., pain, swelling, and/or trismus).

Although the number of studies comparing the earliest versions of PRF with new-generation alternatives is growing each year, direct comparisons between same-generation platelet derivatives (i.e., A-PRF compared with L-PRF or i-PRF) are still scarce due to their relative novelty and lack of inclusion in routine clinical protocols followed in dental surgical procedures. For the most recent platelet-rich fibrins—such as that discussed in this study, A-PRF+—the available data and relevant high-quality randomized clinical trials are even more limited, with the available literature generally focusing instead on laboratory-based results and comparisons between PRF generations. In this systematic review, we included studies that compared A-PRF+ not only with control (e.g., saline) treatments [21,26,29] but also with L-PRF; these comparisons are valuable, even though the results were obtained in relatively small patient groups. Of the cited articles, some did not report any statistically significant differences in the results obtained between A-PRF+ and L-PRF, although it must be noted that different types of platelet-rich fibrin yield different results in different clinical scenarios and, so, the difference in outcome is expected to vary with the used clinical protocol. Additionally, studies have highlighted the importance of user preference (e.g., consistency, malleability, toughness, stickiness, ease of use with grafting material and fixation), as experience and familiarity with the used material plays an important role in the outcome of a surgical procedure [16,30,31]. Although clinical comparisons frequently yield analogous results between the different platelet derivatives, with their differences not reaching statistical significance, it is more important to consider the improvements in clinical outcomes when compared with control treatments (e.g., saline or collagen plug). The results drawn from this systematic review are highly dependent on the available number of studies, the populations (number, ethnicity, age, surgical procedure) enrolled in the comparative studies, and the familiarity of the surgeons with the newest generation of PRFs (and, subsequently, their confidence in handling the material).

The greatest limitations of this study were undoubtedly the relatively small number of articles included and the limited patient sample sizes in the cited articles. The limitation regarding the low number of included studies is due to two major factors. Firstly, the inclusion criteria meant that only randomized clinical trials (RCTs) were included, which had to not only focus on A-PRF+ usage in the healing and soft-tissue regeneration context, but also had to pass the risk of bias assessment (RoS 2). Secondly, another limiting factor was the novelty of A-PRF+ and a general lack of relevant studies, even compared with A-PRF. The relatively limited patient sample sizes included in the systematic review was a consequence of the aforementioned scarcity of available studies that satisfied the rigorous inclusion criteria. Another limitation encountered was the lack of uniform, standardized numerical outcome measures across the included studies—for example, in the assessment of swelling or trismus—which hindered the possibility of making direct comparisons. This further impeded the ability to draw concise conclusions from the extracted results. Despite the extensive search of the available literature on A-PRF+, the articles that met the inclusion criteria were scarce; thus, the different approaches to the same procedures had to be combined for comparison of the outcomes. Otherwise, the possibility of drawing any conclusions would be impossible with such a limited number of available studies. Even though this may affect the strength and reliability of the conclusions—especially compared with further research on the topic—it is still important to establish a starting point for future research. In future reviews, it is expected that the literature on A-PRF+ will be less scarce, allowing more precise conclusions to be drawn. The present study considered A-PRF+ and evaluated its outcomes in comparison to the various control groups in different studies, with the objective of assessing the necessity of subsequent procedures such as the collection of patients’ blood and the utilization of centrifuge equipment, as well as to identify whether the additional requirements outweigh the benefits.

A comprehensive analysis of the results from randomized clinical trial studies revealed the efficacy of A-PRF+ in nearly all categories. Although the results did not always attain statistical significance, they frequently approached the significant *p*-value of *p* = 0.05. Moreover, postoperative pain, swelling, and trismus were notably reduced in patients treated with A-PRF+, leading to an enhancement in their overall quality of life. Furthermore, healing of the postoperative wound and the total reduction in wound area were enhanced when A-PRF+ was utilized in the procedure, with improvements being most noticeable at the 7- and 14-day follow-up visits. This effect was most pronounced in the A-PRF+ study groups during the initial phase of healing, whereas its impact was less evident at later stages. Other variables, such as the presence of inflammation around the postoperative area, epithelialization, bleeding management, and the percentage of incomplete healing, exhibited similar trends and A-PRF+ groups generally outperformed the control groups. The control of postoperative bleeding mentioned in the studies of Brancaccio et al. and Giudice et al. [22,23] indicates that patients undergoing antiplatelet drug therapy can benefit from A-PRF+. These studies presented similar trends even though they were performed in the same research facility by the same team, demonstrating repeatable outcomes in larger study groups. Of the studies reviewed, that of Pereira et al. [25] was the only one that did not observe a positive effect when A-PRF+ was added to the protocol. Considering the effects of A-PRF+ on bone and grafting material behaviors, its net total benefits outweigh the additional cost and steps necessary for its production [10]. This study’s limitations primarily included the low number of research papers that met the inclusion criteria during the literature search and the number of participants in the studies; however, it was considered imperative to base the review on randomized clinical trials, considering the high quality of the results obtained through such studies. It is also noteworthy that the search predominantly focused on postoperative soft tissue management and the postoperative assessment of bleeding, pain, swelling, and trismus, which further restricted the available literature. A-PRF+ is a novel material, even when compared with A-PRF. As time progresses, the number of randomized clinical studies referencing its use can be expected to increase, potentially including larger patient test groups to enhance the statistical significance of the obtained results. Future research should prioritize the inclusion of homogeneous study populations; the rigorous analysis of objective, quantifiable outcomes; and the use of sufficiently powered sample sizes to ensure statistical reliability and generalizability. There is a need to establish uniform standards for the preparation and processing of blood products, as well as for patient selection and qualification. Moreover, surgical protocols and the procedures for preparing both soft and hard tissues should be standardized.

## 5. Conclusions

The following conclusions can be drawn from this systematic review:

- The addition of A-PRF+ can reduce postoperative pain; however, the significance of this effect needs to be verified in future studies.
- The addition of A-PRF+ decreased the postoperative intake of analgesics.
- A-PRF+ addition reduced postoperative swelling and trismus.
- A-PRF+ addition can benefit patients who are undergoing antiplatelet drug therapy, improving postoperative bleeding control.
- Compared to other third-generation platelet-rich fibrins, A-PRF+ did not demonstrate statistically significant differences in terms of clinical outcomes.
- Future research would benefit from the use of consistent measurement methods for the grading of postoperative swelling and trismus, as well as larger patient populations.

## Figures and Tables

**Table 1:** General information about studies included in the review.

No.	Reference	Country	Procedure	No. of Patients	Follow-Up	Complications
1	Alhaj et al. 2018 [21]	Lebanon	Filling the resultant gap after immediate insertion of a mandibular molar implant with A-PRF+ autograft mixture or autograft alone and comparing the outcomes	20	2nd and 7th days; 3 and 6 months	None
2	Brancaccio et al. 2020 [22]	Italy	Extraction of four non-adjacent teeth with treatment using four different hemostatic procedures (sutures only, A-PRF+, hemostatic agent—HEM, or L-PRF)	102	2 weeks	Not specified
3	Giudice et al. 2019 [23]	Italy	Extraction of four non-adjacent teeth with treatment using four different hemostatic procedures (sutures only, A-PRF+, HEM, or L-PRF)	40	1 and 2 weeks	None
4	Kalash et al. 2017 [24]	Lebanon	Immediate implant placement and filling of peri-implant gap with xenograft or PRF–xenograft mixture	18	2nd, 7th, and 14th days; 3, 6, and 9 months	None
5	Pereira et al. 2023 [25]	Brazil	Upper third molar extractions with socket management using A-PRF+ or only blood cloth	16	3rd, 7th, 14th, 30th, and 90th days	Not specified
6	Soto-Penaloza et al. 2019 [26]	Spain	Apical root resection (3 mm) with or without the use of A-PRF+ during free-flap closure	50	7 days	Feeling nauseous, discomfort related to prolonged bleeding, and bad breath/taste
7	Sousa et al. 2020 [27]	Portugal	Patching free gingival graft sites with A-PRF+ clot membranes and evaluating its potential for improving wound healing	25	3 months	Hemorrhage in one control and two test patients (2nd day); one necrosis in control group (7th day)
8	Yewale et al. 2021 [28]	India	Atraumatic tooth extractions and socket preservation with Sybograft plus or Sybograf plus/A-PRF+	20	6 months	None
9	Yüce and Kömerik 2019 [29]	Turkey	Managing alveolar osteitis after third molar extraction using A-PRF+	40	1st, 3rd, 7th, and 15th days; 1, 2, and 3 months	None

Abbreviations: A-PRF+—advanced platelet-rich fibrin plus; HEM—hemostatic agent; L-PRF—leukocyte platelet-rich fibrin.

**Table 2:** VAS pain score and analgesic usage.

No.	Reference	Analgesic Usage (Number of Pills/Day)			VAS Pain Score (Range 0–10)		
1	Pereira et al. 2023 [25]	-			Median (interquartile range)	Control group	A-PRF+ group
					Day 3	1.5 (0–3.75)	2 (0–4.7)
					Day 7	0 (0–2)	0 (0–4.5)
					Day 15	0 (0–0.75)	0 (0–0.75)
2	Soto-Penaloza et al. [26]	** A significant difference in the number of analgesic pills taken per day was not observed between the saline and A-PRF+ groups (*p* > 0.05).				Saline group	A-PRF+
					Day 7	2.07 ± 1.63	1.27 ± 0.85
3	Sousa et al. [27]	-			Median (interquartile range)	Control group	A-PRF+ group
					Day 2 *	2 (2)	0 (1)
					Day 7	1 (2)	0
					Day 14	0	0
					Day 30	0	0
					Day 90	0	0
4	Yewale et al. [28]	-			Pain rate (%)	Control group	A-PRF+ group
					Mild (1–2)	30	20
					Moderate (3–6)	70	80
5	Yüce et al. [29]	* Average after 7 days	Saline group	A-PRF+ group		Saline group	A-PRF+ group
			13.05 ± 1.32	3.6 ± 1.19	Day 0	6.8 ± 0.83	7.15 ± 1.04
					Day 1 *	7.25 ± 1.02	5.2 ± 1.06
					Day 3 *	7.05 ± 1.23	2.25 ± 0.64
					Day 5 *	5.9 ± 0.91	0.8 ± 0.62
					Day 7 *	4.05 ± 0.76	0.45 ± 0.51
					Average	4.43	2.14

Abbreviations: A-PRF+—advanced platelet-rich fibrin plus; VAS—visual analog scale (* *p* ≤ 0.05, ** *p* > 0.05).

**Table 3:** The results of studies reporting on swelling, edema, and trismus.

No.	Reference	Swelling/Edema			Trismus		
1	Alhaj et al. [21]	Median facial swelling (cm)	Saline group	A-PRF+ group	-		
		Day 2	0.36	0.26			
		Day 7	0.11	0			
2	Pereira et al. [25]	Median (interquartile range)	Control group	A-PRF+ group	-		
		Day 2	1.5 (0.25–3.75)	1.5 (0.25–4.5)			
		Day 7	0 (0–1.75)	0 (0–2.75)			
3	Soto-Penaloza et al. [26]	-			Occurrence of trismus (%)	Saline group	A-PRF+ group
						96	68
4	Yewale et al. [28]	Occurrence of swelling (%)	Control group	A-PRF+ group	-		
		Day 3	80	30			
		Day 4	30	0			
		Day 45	50	0			

Abbreviations: A-PRF+—advanced platelet-rich fibrin plus.

**Table 4:** The effects of A-PRF+ addition on wound healing.

No.	Reference	Total Wound Area (mm^2^)			Wound Reduction Area (%)			Visible Epithelialization			Wound Healing					
								(%)								
1	Brancaccio et al. [22]	-			-			-			* Incomplete healing after 2 weeks (%)	Saline	HEM	A-PRF+	L-PRF	
												30	38	22	15	
2	Giudice et al. [23]	-			-			-			Mean Wound healing index	Saline	HEM	A-PRF+	L-PRF	
											** 1 week	1.05 ± 0.6	1.18 ± 0.59	1 ± 0.68	0.95 ± 0.5	
											** 2 weeks	0.33 ± 0.53	0.43 ± 0.5	0.25 ± 0.49	0.15 ± 0.36	
3	Pererira et al. [25]	-			-			-			Median (interquartile range)		Control		A-PRF+	
											Day 3		1.5 (0–5)		3 (0.25–5)	
											Day 7		0 (0–2.75)		1 (0–2.75)	
											Day 15		0 (0–1)		0 (0–1)	
											Day 30		0 (0)		0 (0–0.75)	
											Day 90		0 (0)		0 (0–0.75)	
4	Soto-Penaloza et al. [26]	-			-			-			Inflammation on control (%)		Saline		A-PRF+	
													100		80	
5	Sousa et al. [27]		Control	A-PRF+		Control	A-PRF+		Control	A-PRF+	-					
		** Day 2	119 ± 41.6	118 ± 30.8	** Day 2	2.0 ± 5.1	2.9 ± 10.7	** Day 2	0	0						
		** Day 7	105.1 ± 33.4	77.3 ± 23.3	* Day 7	12.9 ± 12.2	36.4 ± 12.2	** Day 7	0	0						
		** Day 14	74.5 ± 31.9	±50.316.6	* Day 14	36.6 ± 20.4	58.0 ± 14.2	* Day 14	9.1	64.3						
		** Day 30	40.0 ± 17.2	11.0 ± 18.8	* Day 30	50.9 ± 14.3	90.5 ± 14.6	** Day 30	90.9	92.9						
		** Day 100	0	0	** Day 100	100	100	** Day 100	100	100						
6	Yüce et al. [28]	-			-			-			Landry, Turnbull, and Howley Index	Saline	A-PRF+			
											* Day 7	2.05 ± 0.69	3.65 ± 0.49			
											* Day 14	3.55 ± 0.6	4.8 ± 0.41			

Abbreviations: A-PRF+—advanced platelet-rich fibrin plus. (* *p* ≤ 0.05, ** *p* > 0.05).

**Table 5:** Hemostatic effects of A-PRF+.

No.	Reference	Hemostatic Effect				
1	Brancaccio et al. [22]	Bleeding after 30 min (%)	Saline group	Hemostatic group	* A-PRF+ group	* PRF group
			20	12	2	5
2	Giudice et al. [23]	Odds Ratio (OR) after 30 min	Saline group	Hemostatic group	* A-PRF+ group	L-PRF group
			0.25	0.57	0.1	0.21
3	Pereira et al. [25]	Median (interquartile range) at day 3	Control group		A-PRF+ group	
			0.5 (0–2)		1 (0–2)	
4	Soto-Penaloza et al. [26]	Postoperative bleeding (%)	Saline group		A-PRF+ group	
			52		28	

Abbreviations: A-PRF+—advanced platelet-rich fibrin plus (* *p* ≤ 0.05).

## Data Availability

No new data were created or analyzed in this study. Data sharing is not applicable to this article.

## Abbreviations

A-PRF	Advanced platelet-rich fibrin
A-PRF +	Advanced platelet-rich fibrin plus
AFG	Autologous fibrin glue
HEM	Hemostatic agent
I-PRF	Injectable platelet-rich fibrin
IQR	Interquartile range
L-PRF	leucocyte and platelet-rich fibrin
LTH	The Landry, Turnbull, and Howley epithelial healing index
PRF	Platelet-rich fibrin
PRP	Platelet-rich plasma
PROSPERO	Prospective Register of Systematic Reviews
RCTs	Randomized clinical trials
T-PRF	Titanium-prepared platelet-rich fibrin
VAS	Visual analog scale
